# Editorial: Insights in pediatric gastroenterology, hepatology and nutrition: 2022

**DOI:** 10.3389/fped.2023.1210986

**Published:** 2023-05-12

**Authors:** Andrew S. Day

**Affiliations:** Department of Paediatrics, University of Otago Christchurch, Christchurch, New Zealand

**Keywords:** inflammatory bowel disease, Wilson disease, duodenal abnormalities, ankyloglossia, elastography, magnetic resonance imaging

**Editorial on the Research Topic** Insights in pediatric gastroenterology, hepatology and nutrition: 2022

The world of pediatric gastroenterology, hepatology and nutrition continues to expand and evolve over time: 2022 was no exception. Advances included diagnostic approaches, understanding of disease and new therapies. This research topic (RT) aimed to include some of these.

Inflammatory bowel disease (IBD) beginning in childhood is typically more severe and more extensive than onset in adults (1). This is especially so in the subgroup diagnosed prior to their sixth birthday (very early onset IBD). In addition, rates of IBD in children appear to be increasing in many areas of the world (2). Three of the articles included in this RT focused on aspects of IBD in children.

Borowitz provided a focused review of the epidemiology of IBD. As outlined in this review, there are a number of key aspects that likely contribute together to the onset of disease. These include environmental factors, diet, the host microbiome, triggering infections, immune responses and at risk genes. Whilst more is known about these variables, the cause of IBD has not yet been delineated. Further advances may follow clear, focused and detailed epidemiology studies.

Vitamin D is one environmental factor of increasing interest to the epidemiology, pathogenesis and management of IBD (3). Sun et al. explored the some of the relationships between vitamin D in a systematic review. In the 16 studies that met inclusion criteria, the authors determined that there was no difference between vitamin D levels in children with IBD and controls without IBD. The data arising from these reports indicated that vitamin D supplementation can improve disease activity. Further studies focused on vitamin D in children with IBD are required: these could focus further on these relationships, the optimal dosage regimen to maintain vitamin D sufficiency and the treatment target.

Tabari et al., in a third article focusing on IBD in children, compared imaging findings with clinical features in a group of children with fistulising Crohn disease (CD). Thirty-six of the 60 children included were shown to have one or more abscesses associated with their perianal fistula. Magnetic resonance imaging (MRI) findings associated with abscess formation were the length of the fistula tract, and the presence of multiple external openings or multiple fistulae.

The use of MRI for assessment of hepatic fibrosis (MR elastography) has been considered more recently (4). Lorton et al. reported on the development and assessment of a new passive driver for the application of MR elastography in children. The authors determined that their novel driver was considered to be more comfortable and provided enhanced imaging outputs. Further assessment leading to the use of such advances should lead to increased use of MR elastography.

In a second manuscript that focused on an aspect of liver disease in children, Feng et al. described the development of a novel scoring system to enable clinicians to differentiate acute liver failure (ALF) due to Wilson disease from ALF due to other causes. When the use of this scoring system was applied in a cohort of 58 children a cut-off score of ≥6.5 (out of a maximum of 13) provided sensitivity of 91.7% and specificity of 97.8% to identify those with WD (*p* < 0.0001). Inclusion of age further enhanced the utility of the scoring system. This tool now needs further validation in other cohorts with greater patient numbers.

Kroger et al. evaluated the causes and outcomes of non-atrophic duodenal changes in 315 children from a consecutive cohort of 1,107 children undergoing upper gastrointestinal endoscopy in one centre in Finland. There was an association between the finding of non-atrophic changes and anemia, blood loss and abnormal celiac serology tests. The authors showed that, after excluding the subgroup with potential celiac disease, the remainder had good long-term outcomes.

The final manuscript included in this RT provided an overview of the some-times controversial area of ankyloglossia (also known as tongue tie) (5). Borowitz reviewed the literature relating to this condition and noted increasing numbers of children being diagnosed in several countries. The authors also highlighted a number of gaps in the literature including the variety of scoring systems used and the lack of long-term outcome data.

Together these seven reports covered some interesting aspects relevant to the field of pediatric gastroenterology. From these works, a number of questions arise. Hopefully these publications might help to trigger further endeavours that might answer some of the questions posed, and lead on to enhanced management and patient outcomes.
